# Treatment of hypertension and obstructive sleep apnea counteracts cognitive decline in common neurocognitive disorders in diagnosis-related patterns

**DOI:** 10.1038/s41598-023-33701-2

**Published:** 2023-05-09

**Authors:** Milenko Kujovic, Tim Lipka, Mark Zalman, Leonie Baumann, Michaela Jänner, Bruno Baumann

**Affiliations:** 1 Department of Psychiatry and Psychotherapy, Medical Faculty, Heinrich-Heine University Düsseldorf, Düsseldorf, Germany; 2 Department of Neuropsychiatry, Centre for Neurology and Neuropsychiatry, Medical Faculty, Heinrich-Heine University Düsseldorf, Düsseldorf, Germany; 3 Department of Mental Health, University Hospital of Münster, Munster, Germany

**Keywords:** Alzheimer's disease, Hypertension, Dementia, Sleep disorders

## Abstract

The aim of this study was to investigate the effect of arterial hypertension (AH) and of obstructive sleep apnea (OSA) on cognitive course in the neurocognitive disorder (NCD) cohort RIFADE which enrolled patients with NCD due to Alzheimer’s disease (AD), vascular NCD (vNCD), and mixed NCD (AD + vNCD = mNCD). Multiple risk factors (RF), including AH and OSA, that contribute to the development of various kinds of dementia have been identified in previous studies. Studies that observed AH lacked investigation of long-term effects and did not isolate it from other RF. Studies involving OSA as a risk factor did not include participants with all stages of NCD. 126 subjects were screened for AH and OSA. Repeated cognitive measurements were performed with the DemTect as primary outcome and the clock drawing test as secondary outcome measure. 90 patients had AH (71.4%) and 40 patients had OSA (31.7%). RF-status had a significant effect on cognitive outcome in models with RF as single factors (AH p = 0.027, OSA p < 0.001), a 2-factor analysis with AH × OSA (AH as main factor p = 0.027) as well as a model including the 3 factors AH × OSA × diagnosis (p = 0.038). Similarly, a 3-factor model was significant for the clock-drawing test, whereas single factor-models remained insignificant. AH and OSA appear to be risk factors in common NCD and cognitive decline can be mitigated by treatment of these RF.

## Introduction

There is increasing evidence that certain risk factors play a role in the two most common types of dementia, i.e. Alzheimer’s disease (AD) and vascular dementia (VaD). Shown primarily by observational studies, it turned out, that vascular risk factors, hypacusis, environmental conditions and lifestyle behaviors could account for the development of up to 40% of dementias^1^.

This is of particular interest for Alzheimer’s disease (AD), where successful treatments improving or even stabilizing cognitive outcomes for time periods of years have so far been lacking. However, correction or elimination of a suspected pathological factor does not necessarily lead to successful treatment, which has been revealed by numerous attempts to address cellular pathology. Immunotherapies against amyloid or tau pathology have so far failed to show cognitive improvement or stabilization in the long term in AD^2–6^.

As a first important example of such factors contributing to the pathology of neurocognitive disorders (NCD), arterial hypertension (AH) is presented and analyzed in this publication. Being one of the most relevant factors in medicine, AH is clearly proven as a risk factor for Alzheimer´s disease and vascular dementia^7–9^. In addition, there is initial evidence, that correcting elevated blood pressure has positive effects on cognitive outcome^10–13^. However, this has not been shown in the long term and for AH as a single factor, i.e. in an isolated analysis without complexing and merging with other factors^14–16^. In addition, there are few studies addressing the question of whether treatment of AH in patients with a pre-existing NCD could have favorable effects. Wharton et al.^17^ found that participants affected by AH were less likely to convert from MCI to AD when treated with antihypertensive medications. Similar effects were found in participants with already existing AD, usage of antihypertensive medication was associated with a slower rate of cognitive decline compared to AD individuals that had never been treated with this kind of medication^18^.

Another suspected risk factor for dementia is obstructive sleep apnea (OSA). The estimated prevalence of OSA ranges from 9 to 38 percent in the general population. In elderly males, it is estimated at 90 percent^19^. Similarly, a greatly increased odds ratio for developing OSA has been reported with increasing age^20^.

Obstructive sleep apnea is characterized by the collapse of upper airways through relaxed throat musculature causing intermittent hypoxia and sleep fragmentation^21^. For the sleep disturbance to be considered OSA, the breathing cessation must persist for a duration of at least 10 s and occur more than 5 times per hour of sleep. Alternatively, airflow must show a reduction at least five times per hour, including a drop in oxygen or a rise in carbon dioxide^22^. The results of disrupted sleep and hypoxia are excessive daytime sleepiness (EDS)^23^, fatigue, depression^24^, and cognitive complaints^25,26^. Cognitive domains usually impaired in OSA are working memory, vigilance/attention^27^ and executive functioning^19,21,28,29^.

There are several lines of evidence showing OSA as a putative risk factor for neurocognitive disorders including AD. First, the hippocampus as a major target of pathologies leading to dementia, shows high vulnerability to hypoxic events such as those found in OSA^30^. Continuous positive airway pressure (CPAP), the gold standard in the treatment of OSA^31^, seems to have a beneficial influence on mild cognitive impairment (MCI). Improved attention, psychomotor speed and everyday functioning, and reduced EDS have been shown after one year of CPAP^32^. Treating OSA may slow disease progression in MCI patients and even show short-term improvements on cognitive scales^33^. Untreated OSA causes arterial hypertension and is often associated with other vascular risk factors, increasing the risk of cardiac or cerebrovascular diseases potentially leading to dementia^34–36^**.** A long-term follow-up analysis showed a partial remission of cognitive deficits, a reduction of EDS and depressive symptoms^37,38^.

Neuroimaging studies indicated not only functional brain alterations in OSA^39^ but also recovery after CPAP-treatment in brain regions which were affected by hypoxic damage^40^ i.e. the hippocampus^41,42^, the frontal gyri^43^, and the default mode network^44,45^.

Studies including patients already suffering from mild cognitive impairment (MCI) and Alzheimer’s disease showed a benefit for treatment of OSA delaying dementia onset or slowing disease progression^32,37,46–48^. These studies, however, did not investigate the effect of treatment in patient groups including all stages of pre-existing NCD.

Finally, a meta-analysis indicated an epidemiological argument for OSA as a risk factor for AD showing a five-fold increased prevalence of OSA in patients with AD compared to cognitive healthy individuals^49^.

The cohort RIFADE (RIsk FActors of DEmentia) presented here is a single-center cohort with neurocognitive disorder patients enrolled in the lower Rhine area of Germany. This cohort is in detail described in a prior publication^50^. The current study aims to investigate the isolated effects of the risk factors AH and OSA in all stages of NCD.

## Methods

### Study population

The present analysis used the clinical data of the German neurocognitive disorder (NCD) cohort RIFADE (n = 126), which is a retrospective single-center study focusing on the role of risk factors in NCD^50^. Patients of RIFADE entered the study with the diagnoses NCD due to Alzheimer`s disease (AD-NCD), NCD of vascular type (vascular-NCD) or a combination of both diseases (mixed-NCD). Few patients did not fulfill the criteria of one of these disorders, denoted as neurocognitive disorder of unclear etiology (unspecified-NCD). Important exclusion criteria were the presence of severe Parkinson’s disease, frontotemporal degeneration, Lewy-body-disease, and being resident of a nursing home.

Informed consent was obtained from all subjects. The RIFADE cohort is registered on GermanCTR.de with identifier DRKS00027217. It complies with the Declaration of Helsinki and Good Clinical Practice Guidelines and has been approved by The Ethics Committee at the Faculty of Medicine of Heinrich-Heine-University Düsseldorf.

### Assessments

#### Obstructive sleep apnea

The Epworth sleepiness scale was applied to each patient at the first visit. If scores were suspicious for obstructive sleep apnea (score ≥ 10), patients were referred for a polygraphy. In case of an apnea–hypopnea index (AHI) ≥ 5 a polysomnography (PSG) was performed. If PSG indicated a diagnosis of obstructive sleep apnea, an AHI of 5/h was considered as cut-off for the need of treatment according to the criteria of the International Classification of Sleep Disorders^51^. Stages of OSA were classified for severity as mild grade (AHI 5–15), moderate grade (AHI 15–29) and severe grade (AHI ≥ 30).

In cases showing OSA with the need for treatment, continuous positive airway pressure ventilation during sleep (CPAP) was initiated and patients were followed according to local clinical practice. Adherence to treatment by CPAP was defined as a mean use ≥ 4 h per night for > 5 nights per week with a residual AHI < 5/h.

In the cases where CPAP was not tolerated or not possible, another treatment was initiated including the following treatment options: (1) a mandibular advancement device, (2) a positional therapy.

Obstructive sleep apnea (OSA) was considered present in case of the above criteria were met. OSA was considered corrected in case of CPAP or alternative treatment according to the above criteria.

#### Arterial hypertension

Arterial hypertension (AH) was considered present in case of (1) a pre-existing medication with an antihypertensive drug and/or (2) a mean value of blood pressure (BP) > 140/90 mm Hg in at least 10 successive measurements during 5 days and/or (3) an anamnesis indicating existing arterial hypertension.

Arterial hypertension was considered corrected in case of (1) regular intake of at least 1 antihypertensive drug and/or (2) a mean value BP < 140/90 mm Hg in at least 10 successive measurements during 5 days.

#### Primary outcome

As primary outcome variable the DemTect score was assessed at each visit in the patients cohort. Times between visits followed a general scheme of 3, 6, and 12 months after baseline visit followed by yearly visits. According to a natural setting, this scheme varied due to adherence and clinical acuity. The DemTect represents a common cognitive test, which is validated to categorize and predict outcome in NCD^52^. Repeated measurements of DemTect scores were used as absolute scores as well as the change in scores of two neighboured measurements according to the formula: DemTect change = DemTect score at the current measurement minus DemTect score at the previous measurement.

Since the DemTect uses an age-dependent algorithm for the calculation of normalized scores from raw values with a cut-off at the age of 60, there may be comparability problems with repeated measurements in subjects who pass the 60-year limit during observation. Therefore, in these patients the scores derived from the algorithm for subjects aged < 60 (DemTect score < 60) and ≥ 60 years (DemTect score ≥ 60) were averaged at each visit due to the formula: (DemTect score < 60 + DemTect score ≥ 60)/2 in order to achieve age-independent scores for calculation of the DemTect change^53^.

In order to stratify data for the initial stages of the longitudinal cognitive course, initial DemTect scores were referred to groups with initial scores of 13–18 (stage 1), 9–12 (stage 2) and < 9 (stage 3), aiming to achieve a staging similar to that of *subjective cognitive disorder* (stage 1) *mild cognitive impairment* (stage 2), and *dementia* (stage 3). For this staging, the original age-dependent scores of the DemTect were used.

To achieve a measure for the final cognitive outcome in addition to repeated measurements of the primary outcome measure, stages at baseline and stages at the final visit were evaluated for favorable and unfavorable outcomes due to the algorithm: (A) *favorable* outcome was assessed, if patients started (1) in stage 1 or 2 and remained in the same stage, (2) started in stage 2 and improved to stage 1, (3) started in stage 3 and improved to stage 1 or 2. (B) *Unfavorable* outcome was assigned to all other cognitive courses, which were different from A.

As a secondary outcome, Shulman’s clock-drawing test was performed^54^. This brief screening test relying on visuo-constructive abilities has proven to reliably discriminate between patients suffering from Alzheimer’s disease, mild cognitive impairment and healthy individuals^55,56^. Testing intervals were the same as for the DemTect (3, 6, 12 months and yearly follow up visits).

#### Evaluation of risk factors

To study the influence of risk factors on the cognitive outcome, neurocognitive time periods (NCT) between 2 successive measurements of the primary outcome variable were established. Since the primary outcome variable was recorded as repeated measures, each patient exhibits at least 1 NCT. The risk factors AH and OSA were evaluated for each NCT regarding (1) presence status, (2) correction status, according to the above-mentioned criteria. If the time of correction of a risk factor exceeded 50% of NCT, the correction status for this factor in this NCT was assigned as "corrected", otherwise the correction status was recorded as "uncorrected". For more details see the previous publication on RIFADE^50^.

Both types of risk factor status, presence and correction status, were integrated into a three-fold status, i.e. the status "absent" (A), if the risk factor is not present, "treated" (T+), if the factor is considered corrected due to the criteria given above, "untreated" (T−), if the factor is not considered corrected.

### Statistics

The relationships between DemTect score/DemTect change and the status of AH and OSA were investigated by mixed effects repeated measurement models. For this purpose, the risk factors AH and OSA as well as the combination thereof were taken as a fixed effect and extra variability in repeated measurements originating from individual patients was taken as a random effect. Different models according to possible combinations of risk factors were calculated and corrected for the parameters age, DemTect at baseline, education and time since inclusion or time to measurement (NCT). In addition, a model with diagnosis as a further factor was performed. According to the analysis performed for the DemTect as the primary outcome all four models/linear mixed models were calculated for the clock-drawing test/secondary outcome as well.

χ^2^-tests are calculated to examine the influence of treated risk factors on favourable cognitive outcome. T-tests are used to compare the observation time of the treated and untreated groups with respect to the risk factors.

Analysis was performed with SPSS 26. N = 126 patients were observed.

### Ethical considerations

This study was designed and conducted according to the Declaration of Helsinki. The study protocol was approved by the Ethical Committee of the Heinrich Heine University Düsseldorf. The study was performed under the laws of General Data Protection Regulation (GDPR) and the Code of Good Conduct.

## Results

### Demographic and clinical data

**Figure 1:**
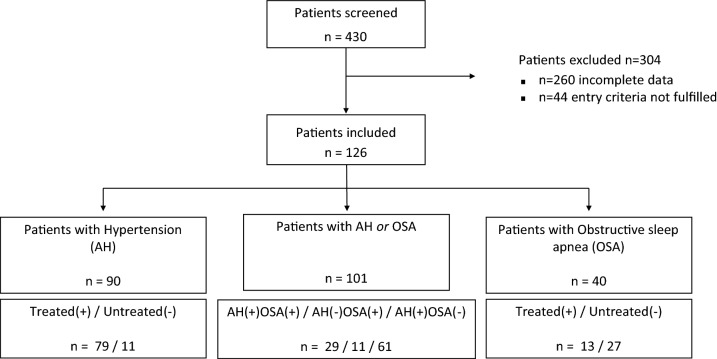
Flow chart patient inclusion. AH, arterial hypertension; OSA, obstructive sleep apnea; Treated(+), risk factor is permanently treated during patient observation; *Untreated(−)*, risk factor is not treated in at least 1 neurocognitive time period (NCT) of patient observation.

All patients of the RIFADE cohort were included in this study (n = 126). According to the above mentioned criteria, 90 patients had arterial hypertension (AH) in this cohort (71.4%) and 40 (31.7%) appeared to have obstructive sleep apnea (OSA). Numbers of combinations of AH and OSA are shown in the flow chart (Fig. 1). Seventy nine of the AH patients had permanently corrected hypertension (87.8%) and 13 of the OSA patients had permanent OSA treatment (33.3%). 11 of the 13 permanently treated patients with OSA received CPAP treatment, one patient used a mandibular advancement device, one patient performed position therapy. Four patients with OSA received treatment non-permanently, i.e. not in all NCTs.

Patients with AH were older, more often male and had a lower score in the DemTect at baseline compared to those without AH. No differences were present in the level of education and comorbidity with OSA between these groups.

Patients with OSA were more often male compared to gender distribution across groups. Patients in the treated group were higher educated, younger, had a higher score in the DemTect baseline, and had a lower proportion of vascular NCD as compared to the untreated group. T-Tests revealed only significant differences for the covariate age since the AH+ (p = 0.0001) and OSA− (p = 0.0002) groups were older than their counterparts. Demographic and clinical data are shown in Tables 1 and 2.

**Table 1:** Demographic data of patients with and without arterial hypertension.

Demography	Missings	Total (n = 126, 100%)	AH− (n = 36, 28.6%)	AH+ (n = 90, 71.4%)	T-test/χ^2^-test	p-value
Mean age (yrs, SD)	0	70.60 ± 10.61	65.31 ± 11.38	72.71 ± 9.49	t(124) = − 3.730	0.0001
Female sex (n, %)	0	66 (52.4%)	22 (61.1%)	44 (49%)	χ^2^(1) = 1.540	0.215
Education (yrs, SD)	0	9.25 ± 2.53	9.25 ± 2.1	9.26 ± 2.69	t(124) = − 0.020	0.492
DemTect baseline	0	12.64 ± 4.24	13.40 ± 4.12	12.33 ± 4.27	t(124) = 1.283	0.201
OSA present (n, %)	0	40 (31.7%)	11 (30.6%)	29 (32.2%)	χ^2^(1) = 0.033	0.856
Diagnosis (n)	0	80/28/7	22/7/2	58/21/5	χ^2^(3) = 1.756	0.624

*AD* Alzheimer’s disease, *AH* arterial hypertension, *Diagnosis* patients with the neurocognitive disorder (NCD) of mixed/vascular/Alzheimer type (unspecified omitted)*, n* number of subjects, *SD* standard deviation, +*/−* with/without AH, *yrs* years.

**Table 2:** Demographic data of OSA patients.

(a) Obstructive sleep apnea patients divided by Apnea–Hypopnea Index^a^					
Demography	Missings	Total (n = 40, 100%)	OSA mild (n = 11, 27.5%)	OSA moderate (n = 14, 35%)	OSA severe (n = 15, 37.5%)
Mean AHI	0	25.34 ± 14.96	9.6 ± 3.69	20.45 ± 4.07	40.97 ± 11.11
Female sex (n, %)	0	11 (27.5%)	5 (45%)	1 (7.1%)	5 (33%)
Education (yrs, SD)	0	10.25 ± 3.24	9.25 ± 2.1	9.26 ± 2.69	9.26 ± 2.69
Mean age (yrs, SD)	0	68.54 ± 11.10	72.92 ± 8.65	65.17 ± 11.05	68.47 ± 11.62
DemTect baseline	0	13.1 ± 3.79	14.21 ± 3.32	12.67 ± 4.53	12.28 ± 2.80
BMI	11	27.27 ± 3.97	26.76 ± 4.45	26.96 ± 3.28	28.83 ± 3.72
Diagnosis (n)	0	20/11/1	6/2/0	4/6/0	10/3/1
AH present (n, %)	0	29 (72.5%)	9 (82%)	9 (64.3%)	11 (73.3%)

(b) Obstructive sleep apnea patients divided by treatment status^b^					
Demography	OSA+ (n = 13)	OSA− (n = 27)	T-test/χ^2^-test	p-value	
Mean AHI	29.175 ± 14.59	23.82 ± 14.9	t(38) = 1.071	0.290	
Female sex (n, %)	4(31%)	6(22%)	χ^2^(1) = 0.342	0.559	
Education (yrs, SD)	11.15 ± 3.30	9.81 ± 3.12	t(38) = 1.249	0.219	
Mean age (yrs, SD)	60.12 ± 11.63	72.59 ± 8.17	t(38) = − 3.929	0.0002	
DemTect baseline	14.31 ± 4.16	12.52 ± 3.46	t(38) = 1.343	0.160	
BMI	27.57 ± 3.46 (2m)	27.08 ± 4.27 (9m)	t(27) = 0.321	0.751	
AH present	9(69%)	20(74%)	χ^2^(1) = 0.103	0.748	
Diagnosis	7/1/1	13/9/0	χ^2^(1) = 5.027	0.151	

^a^*AHI* Apnea–Hypopnea Index*, BMI* body mass index*, Diagnosis* see Table 1, *moderate* AHI 15–29.9, *n* number of subjects*, OSA* obstructive sleep apnea, *SD* standard deviation, *severe* AHI ≥ 30, *mild* AHI 5–14.9, *yrs* years.

^b^*BMI* Body Mass Index, *Diagnosis* see Table 1, *m* missings*, OSA-* untreated OSA patients who were either CPAP incompliant (n = 6), or received CPAP therapy for < 50% of total NCT (n = 4), or did not receive any OSA therapy according to medical records (n = 17), *OSA* + treated OSA patients, *yrs* years.

### Mixed model analysis

**Figure 2:**
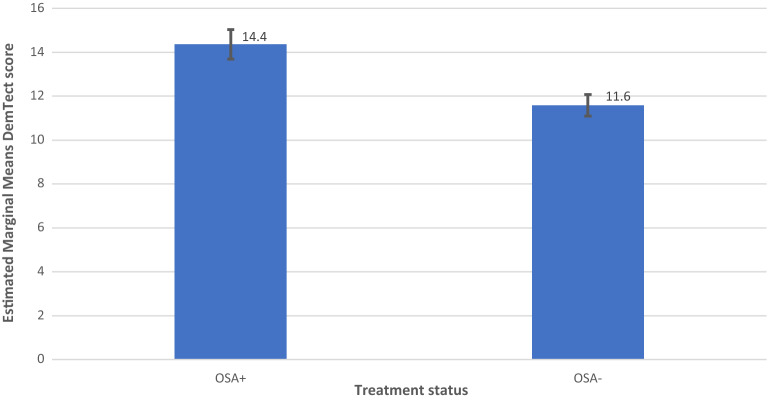
Model 2: OSA as Single Factor, including M and SE.

**Figure 3:**
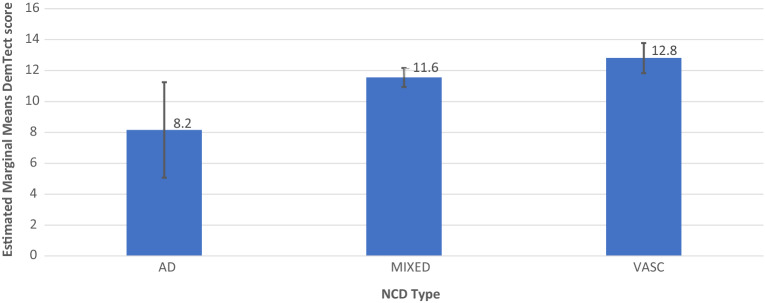
Model 4 AH × OSA × Diagnosis for AH+OSA− including M and SE.

**Figure 4:**
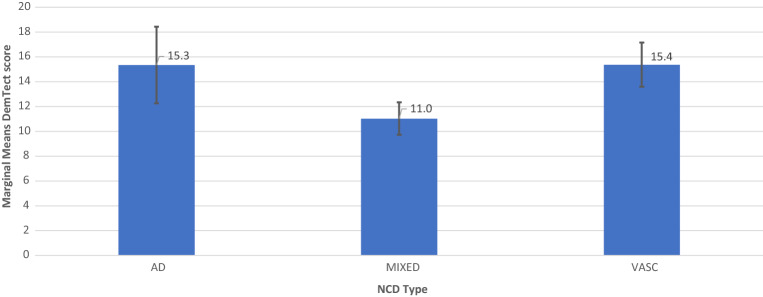
Model 4 AH × OSA × Diagnosis for AH+OSA+ DemTect score, including M and SE.

**Figure 5:**
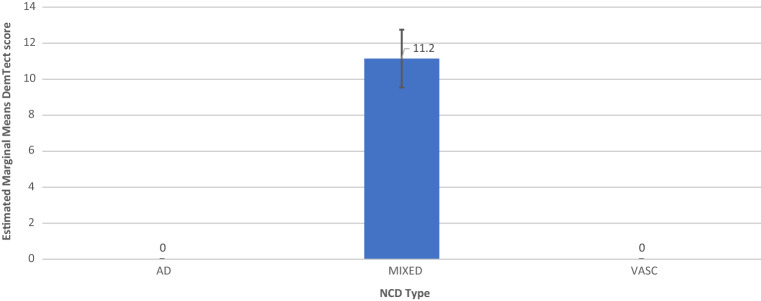
Model 4 AH × OSA × Diagnosis for AH−OSA− including M and SE.

**Figure 6:**
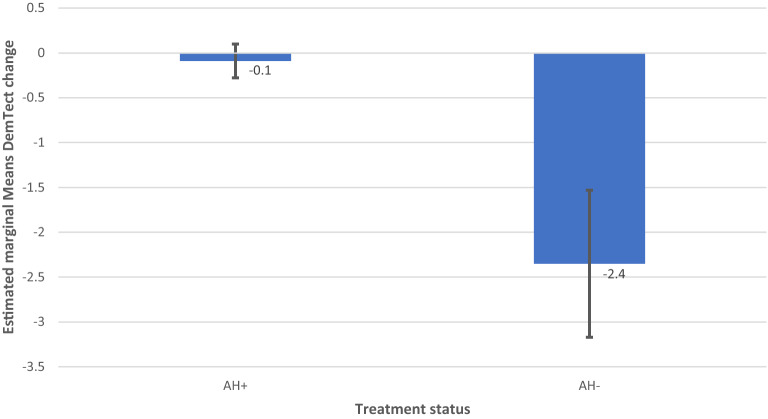
Model 1 AH as Single Factor DemTect change including M and SE.

**Figure 7:**
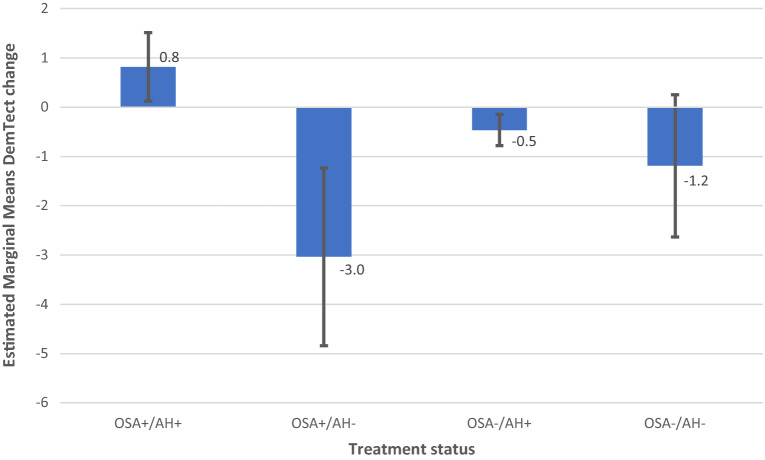
Model 3 AH × OSA DemTect change including M and SE.

In order to analyze the relationships between DemTect score/DemTect change and AH- and OSA-status, 4 models were run as mixed linear models. In addition to the analysis of the single factors AH (model 1) and OSA (model 2), the combination of both factors (model 3) and a model additionally including diagnosis as factor (model 4) was calculated. Each model was corrected for the parameters age, time since inclusion (for DemTect score) or time to measurement (NCT) for DemTect change, DemTect score at baseline and education. For analyses which included diagnosis as a factor, patients with NCD-unspecified were excluded from the analysis. Results are shown in Tables 3 and 4 and Figs. 2, 3, 4, 5, 6 and 7. Further details can be found Supplementary Tables 1–5 .

**Table 3:** Mixed linear model analysis of repeated DemTect scores as dependent variable with (a) fixed effects type III, and (b) mean values for risk factors in different states.

(1a) Model 1: AH as single factor^A^						
Source		Numerator-df		Denominator-df	F	p
Constant term		1		140.649	29.918	0.000
AH		2		212.023	1.583	0.208
Age (yrs)		1		160.070	15.972	0.000
Education		1		120.031	0.208	0.649
Time		1		356.930	24.515	0.000
DemTect baseline		1		119.459	123.074	0.000

(1b) Model 1: AH as single factor^B^						
AH		M	SE	Df	Confidence interval 95%	
					Lower bound	Upper bound
Absent		12.026^a^	0.484	131.536	11.067	12.984
Treated		11.820^a^	0.296	133.749	11.235	12.406
Untreated		10.337^a^	0.877	358.256	8.611	12.062

(2) Model 2: OSA as single factor^C^						
Source		Numerator-df		Denominator-df	*F*	*p*
Constant term		1		127.529	26.073	0.000
OSA		2		205.267	8.507	0.000
Age (yrs)		1		149.177	7.405	0.007
Education		1		113.613	1.252	0.265
Time		1		352.392	37.170	0.000
DemTect baseline		1		111.640	115.519	0.000

(3a) Model 3: AH × OSA^D^						
Source		Numerator-df		Denominator-df	F	p
Constant term		1		134.739	27.847	0.000
AH		2		180.897	2.735	0.068
OSA		2		256.012	1.475	0.231
AH × OSA		4		256.666	1.689	0.153
Age (yrs)		1		153.478	10.631	0.001
Education		1		115.844	0.953	0.331
Time		1		354.551	37.306	0.000
DemTect baseline		1		114.613	122.135	0.000

(3b) Model 3: AH × OSA^E^						
OSA	AH	M	SE	Df	Confidence interval 95%	
					Lower bound	Upper bound
Absent	Absent	11.222^a^	0.573	144.825	10.089	12.354
	Treated	11.553^a^	0.363	140.246	10.834	12.271
	Untreated	9.974^a^	1.213	365.969	7.589	12.359
Treated	Absent	15.489^a^	0.931	206.883	13.654	17.325
	Treated	13.237^a^	0.988	150.161	11.286	15.188
	Untreated	9.328^a^	2.269	173.977	4.850	13.806
Untreated	Absent	11.562^a^	0.951	244.244	9.690	13.435
	Treated	11.855^a^	0.579	132.295	10.710	12.999
	Untreated	11.391^a^	1.439	365.788	8.562	14.221

^A^*AH* arterial hypertension in the states absent, treated, untreated, *DemTect baseline* DemTect score in initial testing, *Denominator df* = denominator degrees of freedom, *Education* Education in the categories low/intermediate/high, *Numerator df* numerator degrees of freedom, *Time* time since inclusion.

^B^Note: ^a^Covariates in the model were calculated using: Age 72.932, Education 1.45, Time since inclusion 973.1284, DemTect score 11.993. *Absent* risk factor (RF) is not present, *treated* RF is present and treated, *untreated* RF is present and untreated, *AH* arterial hypertension in the states absent, treated, untreated, *SE* Standard error.

^C^*OSA* obstructive sleep apnea in the states absent, treated, untreated. For further abbreviations see Table 3(1a) and (1b).

^D^For abbreviations see Table 3(1a) and (1b).

^E^Note: ^a^Covariates in the model were calculated using: Age 72.932, Education 1.45, Time since inclusion 973.1284, DemTect score baseline 11.993. For other abbreviations see Table 3(1a) and (1b).

**Table 4:** Mixed linear model analysis of DemTect change as dependent variable with (a) fixed effects type III and (b) mean values for risk factors in different states.

(1) Model 1: AH as single factor^A^							
Source		Numerator-df		Denominator-df		F	p
Constant term		1		366		12.748	0.000
AH		2		366		3.642	0.027
Age (yrs)		1		366		10.589	0.001
Education		1		366		0.258	0.612
NCT		1		366		16.582	0.000
DemTect start		1		366		5.487	0.020

(2a) Model 2: OSA as single factor^B^							
Source		Numerator-df		Denominator-df		F	p
Constant term		1		366		12.041	0.001
OSA		2		366		1.287	0.277
Age (yrs)		1		366		6.094	0.014
Education		1		366		0.355	0.551
NCT		1		366		19.017	0.000
DemTect start		1		366		5.049	0.025

(2b) Model 2: OSA as single factor^C^							
OSA		M	SE	Df		Confidence interval 95%	
						Lower bound	Upper bound
Absent		− 0.147^d^	0.201	366		− 0.542	0.248
Treated		0.409^d^	0.478	366		− 0.531	1.348
Untreated		− 0.480^d^	0.286	366		− 1.042	0.082

(3) Model 3: AH × OSA^D^							
Source		Numerator-df		Denominator-df		F	p
Constant term		1		366		12.194	0.001
AH		2		366		3.638	0.027
OSA		2		366		0.158	0.854
AH × OSA		4		366		0.550	0.699
Age (yrs)		1		366		8.598	0.004
Education		1		366		0.263	0.608
NCT		1		366		17.211	0.000
DemTect start		1		366		5.776	0.017

(4a) Model 4: AH × OSA × diagnosis^E^							
Source		Numerator-df		Denominator-df		F	p
Constant term		1		366		15.154	0.000
Diagnosis		3		366		1.221	0.302
AH		2		366		2.964	0.053
OSA		2		366		1.514	0.221
AH × OSA		4		366		0.308	0.873
Diagnosis × AH		4		366		0.400	0.809
Diagnosis × OSA		6		366		1.588	0.149
Diagnosis × AH × OSA		2		366		2.076	0.127
Age (yrs)		1		366		9.503	0.002
Education		1		366		0.023	0.879
NCT		1		366		20.260	0.000
DemTect start		1		366		8.654	0.003

(4b) Model 4: AH × OSA × diagnosis^F^							
OSA	AH	Diagnosis	M	SE	Df	Confidence interval 95%	
						Lower bound	Upper bound
Absent	Absent	AD	− 0.443^d^	1.405	366	− 3.207	2.320
		MIXED	− 0.430^d^	0.422	366	− 1.260	0.400
		VASC	1.252^d^	1.089	366	− 0.890	3.394
	Treated	AD	− 0.023^d^	0.985	366	− 1.959	1.913
		MIXED	− 0.117^d^	0.264	366	− 0.637	0.402
		VASC	0.948^d^	0.636	366	− 0.303	2.198
	Untreated	AD	^c,d^				
		MIXED	− 2.202^d^	1.256	366	− 4.672	0.268
		VASC	^c,d^				
Present	Absent	AD	^c,d^				
		MIXED	0.379^d^	0.861	366	− 1.313	2.071
		VASC	− 0.070^d^	0.917	366	− 1.874	1.733
	Treated	AD	7.622^d^	2.810	366	2.095	13.148
		MIXED	− 0.589^d^	0.913	366	− 2.385	1.207
		VASC	2.244^d^	1.175	366	− 0.067	4.554
	Untreated	AD	^c,d^				
		MIXED	^c,d^				
		VASC	^c,d^				
Untreated	Absent	AD	^c,d^				
		MIXED	− 1.366^d^	1.035	366	− 3.402	0.670
		VASC	0.827^d^	0.994	366	− 1.128	2.781
	Treated	AD	− 4.446^d^	2.809	366	− 9.970	1.078
		MIXED	− 0.514^d^	0.344	366	− 1.191	0.163
		VASC	− 0.058^d^	0.729	366	− 1.492	1.376
	Untreated	AD	^c,d^				
		MIXED	− 1.895^d^	1.626	366	− 5.093	1.302
		VASC	^c,d^				

^A^*DemTect start* DemTect score at begin of NCT, *NCT* time between repeated measurements of the DemTect. For other abbreviations see Table 3(1a) and (1b).

^B^For abbreviations see Tables 3(1a) and (1b) and 4(1).

^C^For abbreviations and notes see Tables 3(1a) and (1b) and 4(1).

^D^For abbreviations see Tables 3(1a) and (1b) and 4(1).

^E^For abbreviations see Tables 3(1a) and (1b), 4(1) and Supplementary Tables 2, 3.

^F^For abbreviations and notes see Tables 3(1a) and (1b), 4(1) and Supplementary Tables 2, 3.

Model 1 revealed a non-significant effect of AH on DemTect scores (F (2, 212.023) = 1.583 p = 0.208) and a significant effect of AH on DemTect change (F (2, 366) = 3.642, p = 0.027).

Model 2 showed a significant effect of OSA on DemTect scores (F (2, 205.267) = 8.507, p = 0.0003) and a non-significant effect of OSA on DemTect change (F (2, 366) = 1.287, p = 0.277).

In model 3, 2-factorial analysis with the predictors OSA and AH revealed a non-significant effect of AH (F (2, 180.897) = 2.735, p = 0.068) and of OSA and OSA x OH (F (2, 256.012) = 1.475, p = 0.231) and (F (4, 256.666) = 1.689, p = 0.153) on DemTect scores. The same analysis resulted in a significant effect of AH (F (2, 366) = 3.638, p = 0.027) and a non-significant effect of OSA (F (2, 366) = 0.158, p = 0.854) and AH × OSA (F (4, 366) = 0.55, p = 0.699) on DemTect change.

**Figure 8:**
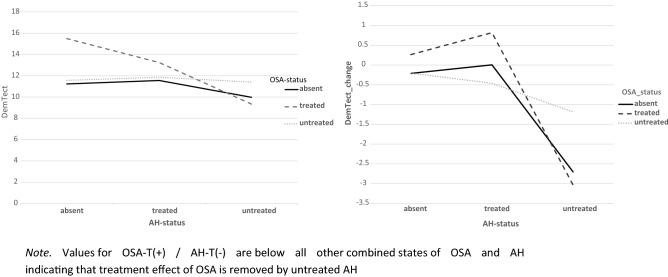
Synopsis of mean DemTect scores (l) and mean DemTect change (r) derived from model 3 for different states of OSA and AH. N*ote.* Values for OSA-T(+)/AH-T(−) are below all other combined states of OSA and AH indicating that treatment effect of OSA is removed by untreated AH.

A synopsis of mean DemTect scores as revealed in model 3 for different states of OSA and AH is shown in Fig. 8.

Model 4 showed a significant interaction effect of all 3 factors AH × OSA × diagnosis on DemTect scores (F (2, 202.324) = 3.329, p = 0.038) and a significant main effect of OSA on DemTect scores (F (2, 262.129) = 6.141, p = 0.002) and a non-significant main effect of AH on DemTect change (F (2, 366) = 2.964, p = 0.053). Diagnosis revealed a non-significant effect on DemTect scores (F (2, 123.538) = 2.941, p = 0.057). All other analyses in this model proved non-significant for single or combined factors on both outcome variables.

In order to analyze the effects of different states of risk factors AH and OSA in diagnostic groups, two separate models were calculated with AH and diagnosis (model 5) and OSA and diagnosis (model 6) as factors and DemTect change as the dependent variable, including the same parameters as covariates as in the above-reported models. Whereas model 5 showed no significant effect on DemTect change, neither for AH as a single factor, nor for the combination AH x diagnosis, model 6 revealed a significant effect of OSA (F(2, 357) = 5.16, p = 0.006) and a non-significant effect of OSA x diagnosis (F(4,357) = 2.20, p = 0.068). Effects of diagnosis in different states of risk factors could be evaluated by estimated marginal means to give an impression of diagnosis-related patterns (see Table 5 and Supplementary Table 6).

**Table 5:** Estimated marginal means of DemTect change as dependent variable in (a) AH × diagnosis, and (b) OSA × diagnosis.

(a) Diagnosis × AH^A^						
Diagnosis	AH	M	SE	Df	Confidence interval 95%	
					Lower bound	Upper bound
AD	Absent	− 0.356^e^	1.438	357	− 3.185	2.472
	Treated	0.338^e^	0.901	357	− 1.433	2.110
	Untreated	^c, e^				
MIXED	Absent	− 0.391^e^	0.377	357	− 1.131	0.350
	Treated	− 0.281^e^	0.207	357	− 0.689	0.127
	Untreated	− 2.046^e^	1.017	357	− 4.047	− 0.046
VASC	Absent	0.525^e^	0.596	357	− 0.647	1.697
	Treated	0.694^e^	0.458	357	− 0.208	1.595
	Untreated	^c, e^				

(b) Diagnosis × OSA^B^						
Diagnosis	OSA	M	SE	Df	Confidence interval 95%	
					Lower bound	Upper bound
AD	Absent	− 0.134^e^	0.821	357	− 1.747	1.480
	Treated	7.429^e^	2.843	357	1.838	13.021
	Untreated	− 4.618^e^	2.842	357	− 10.208	0.971
MIXED	Absent	− 0.287^e^	0.218	357	− 0.715	0.141
	Treated	0.045^e^	0.642	357	− 1.218	1.308
	Untreated	− 0.627^e^	0.321	357	− 1.259	0.004
VASC	Absent	0.959^e^	0.563	357	− 0.148	2.066
	Treated	0.820^e^	0.733	357	− 0.622	2.262
	Untreated	0.220^e^	0.595	357	− 0.950	1.391

^A^Note: ^e^Covariates in the model were calculated using: Age 73.505, Education 1.44, Time between measurements/neurocognitive time (NCT) 415.3361, DemTect score baseline 11.903. For other abbreviations see Tables 3(1a) and (1b) and Supplementary Tables 2, 3.

^B^For other abbreviations see Tables 3(1a) and (1b), 5(a) and Supplementary Tables 2, 3.

**Figure 9:**
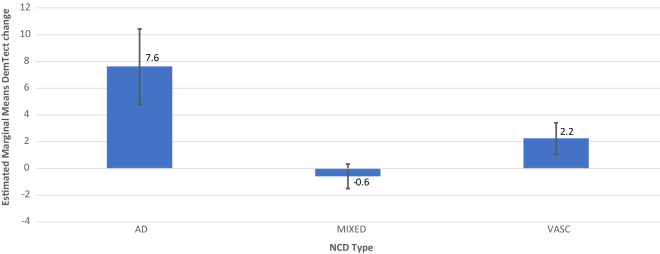
Model 6 AH × OSA Diagnosis OSA+ AH+ DemTect change including M and SE.

**Figure 10:**
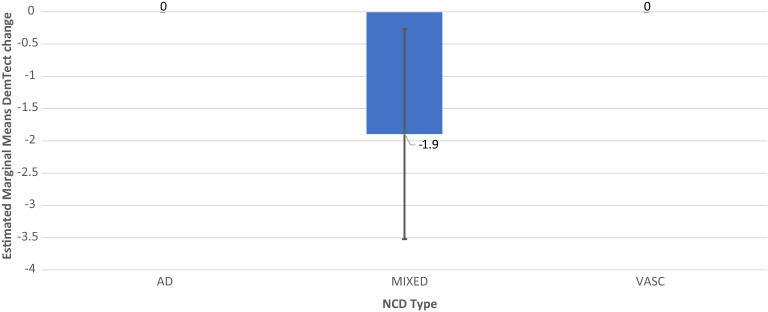
Model 6 AH x OSA Diagnosis OSA− AH− DemTect change including M and SE.

**Figure 11:**
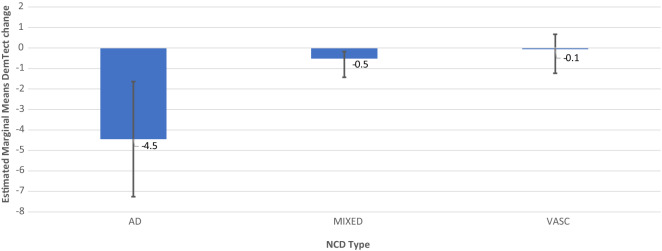
Model 6 AH x OSA Diagnosis OSA− AH+ DemTect change including M and SE.

In all diagnostic groups, i.e. AD-NCD, vascular-NCD, and mixed-NCD, DemTect change is best, if OSA is treated and DemTect change is better in treated compared to untreated groups with regard to OSA or AH. Even if the untreated condition is not observed, as is the case with AH in AD-NCD and vascular-NCD, DemTect change is better in the treated state compared to the absent state. However, whereas in AD-NCD and vascular-NCD the combination of treated OSA and treated hypertension achieves the best results, in mixed-NCD this is the case in the combination with absent AH. On the other hand, worst outcomes are obtained in combinations with untreated OSA in AD-NCD and vascular-NCD, but in mixed-NCD this appears for the combination of absent OSA and untreated AH (see Figs. 8, 9, 10, 11 and Supplementary Table 6).

Model 4 showed a significant interaction effect of all 3 factors AH × OSA × diagnosis on clock-drawing test scores (F (2, 210.670) = 3.432, p = 0.034) whereas OSA and AH as single factors remained insignificant. Time (F (1, 329) = 43.1197, p = 0.000) and clock-drawing test results baseline (F (1, 116.259) = 100.588, p = 0.000) were found to be significant covariates in the model.

### Ancillary analysis

In order to analyze risk factors on a patient level, subjects were divided into permanently corrected hypertension (n = 79) and non-permanently corrected hypertension (n = 11). Whereas 50 of the permanently corrected patients had a favorable cognitive outcome, this was only the case in 3 of the non-permanently corrected patients. A significant association could be found between this assignment and the development of the clinical stage (favorable or unfavorable) from baseline to last observation (Fisher-test: χ^2^ = 5.17, p = 0.045). There was no significant difference between the mean observation time in both groups (t(124) = − 0.145, p = 0.885).

Similarly, patients with OSA were divided into permanently corrected OSA (n = 13) and non-permanently corrected OSA (n = 27). Here, no association could be found between corrected and non-corrected OSA with regard to favorable vs non-favorable outcome (Fisher-test: χ^2^ = 3.25, df = 1, p = 0.178). There was also no significant difference between the mean observation time in both groups (t(38) = 0.412, p = 0.682).

An analysis of diagnostic attributions in the non-corrected, i.e. the untreated group of OSA patients with regard to outcome revealed, that 9 of 10 untreated OSA patients with vascular-NCD, but only 8 of 17 untreated OSA patients with mixed-NCD showed a favorable cognitive outcome (Fisher-test:χ^2^ = 4.98, df = 1, p = 0.042).

To obtain data regarding hypertension in the elderly, patients with treated hypertension aged ≥ 80 years were compared to those with treated AH aged < 80 years. When covaried for DemTect baseline, these comparisons did not reveal a significant difference in outcomes, neither for DemTect scores (F(1, 75.489), p = 0.273) nor for DemTect change (F(1,255), p = 0.508).

## Discussion

The major objective of this study was to evaluate the effect of treatment for arterial hypertension (AH) and obstructive sleep apnea (OSA) on cognitive decline in elderly patients from a cohort with all stages of common neurocognitive disorder. Our major findings are the following. AH and OSA as assessed for the states absent, treated and untreated predict cognitive course as analyzed by DemTect scores or their change and sufficient treatment of each factor results in a positive effect on the cognitive course. When both factors are investigated together, OSA turns out as a significant factor and a non-significant interaction of OSA with AH is revealed. The 3 factors AH, OSA and diagnosis predict cognitive outcome as assessed by DemTect scores.

For the clock-drawing test diagnosis, AH and OSA status revealed a significant interaction effect.

The harmful interplay/additive effect of the investigated risk factors and underlying pathology may worsen cognitive outcome/visuo-constructive skills as measured by the clock-drawing test. Since AH and OSA as single risk factors were not sufficient to predict cognitive outcome this test might be less sensitive than the DemTect.

In a COPD cohort worse DemTect scores were linked to oxygen saturation parameters confirming its sensitivity for detecting cognitive changes due to hypoxic states which are also characteristic for OSA^53^.

### Arterial hypertension

Hypertension reveals an effect on the cognitive outcome as presented by the change in DemTect scores. Here AH reveals a significant effect in both the single factor analysis as well as in the 2-factor statistical approach with AH and OSA as independent variables. Significance disappears for AH when diagnosis as a factor is included in the model. Detailed analyzes of estimated marginal means show an effect of hypertension in all diagnostic groups with a more profound effect in mixed than in vascular NCD (Table 5). Since none of the patients in the vascular group had uncorrected hypertension, this missing data may explain the loss of significance in the analysis including diagnosis as a factor, where a level of p = 0.053 is obtained for AH as a factor in this model.

The results showing an effect of blood pressure (BP) on cognitive outcome are in line with reports of high BP resulting in faster cognitive decline as compared to borderline BP or normal BP in subjects without pre-existing NCD^11^. As shown by the current results, it should be noted that treatment of blood pressure reduces cognitive decline also in individuals *with* NCD. There are only few reports on the comparison of treated vs untreated hypertension in subjects with NCD. A Brazilian study analyzed the effects of the antihypertensive calcium channel antagonist (CCA) nimodipine as a co-medication in mixed dementia showing no effect of nimodipine on psychomotor speed or quality of life. Although using an RCT design, this study gives no information about the treatment status of hypertension given by other antihypertensive agents and their effect on cognitive outcomes^57^. A study on AD patients responding to cholinesterase inhibitors demonstrated an independent effect of antihypertensive treatment in this subgroup over 40 weeks^58^. A further study on the CCA nilvadipine demonstrated significant effects on cognitive measures in AD patients over 6 weeks^59^. In a French cohort, AD patients treated with renin-angiotensin system (RAS) acting medications had slower long-term cognitive decline than those without such medication^17^. Whereas hypertension status in this study appears sure in RAS-users, the compared group of non-users might include patients without hypertension.

In sum, most of the few studies on the treatment of hypertension in subjects with NCD reveal a favorable effect on cognitive outcome up to a time period of less than 1 year. For the first time, the current study demonstrates favorable results of BP correction in NCD patients in the long term.

In the current study, patients with hypertension aged over 80 years had no different cognitive outcome of antihypertensive treatment than those younger than 80 years. This is in conflict with reports of a failing or even worsening cognitive effect of lowering BP in the elderly^60–63^, contrasting with positive results in middle-aged subjects^64^. Possible reasons for the positive results of lowering BP in older patients in RIFADE could be the following: (1) In RIFADE the coupling of the cognitive outcome as assessed by repeated measurements in neurocognitive time periods (NCT) with the concomitant status of treated or untreated hypertension in each NCT allows for evaluation of medium-term effects of treatment and not only of long-term effects on cognition. The analysis of favorable outcome in the long-term in the current study also shows an effect of lowering BP in older individuals, indicating that the positive results in mixed linear models may not be solely due to medium-term effects. Thus, positive results might be obtained due to methodological reasons by analysis of different time windows. (2) RIFADE investigates subjects with preexisting NCD. Positive results are in line with reports showing an increased risk for dementia with high BP in subjects with MCI^65^. (3) No subjects over 90 years old were included in RIFADE. Therefore, no data are available in this study about nonagenarians or centenarians. Moreover, it is to consider that with preceding historical times and improving treatments for cardiovascular diseases, lifespan expectations might be extended and biological aging retarded. Therefore, the octogenarians of the 1990s years for example might be comparable to the nonagenarians of the 2010s years, which may further complicate comparisons of the current data with previous reports. (4) Reports of a raising systolic BP in subjects with NCD up to the age of 80 years with decreasing values only in those over 80 years^66^ support the importance of this observation in RIFADE and suggest that when BP is elevated in elderly patients, should also be lowered in these patients. (5) For individuals older than 90 years, other pathogenetic profiles may exist and other guidelines for BP regulation might be needed.

There are several pathophysiological assumptions about how hypertension might affect cognition. A central role is seen in mitochondrial dysfunction resulting into dysregulation of cellular redox condition and reduced cell survival^67^. Since tau phosphorylation is involved in mitochondrial functioning, it is conceivable, that hypertension, as a classical vascular risk factor, could also be linked to Alzheimer’s pathology^8,9^. This is also supported by recent reports of greater amyloid deposition in the presence of hypertension in middle-aged subjects^68^. A broader and unifying concept of how elevated BP affects cognition brings the link between vasculature and neural function to the fore in terms of the neurovascular unit^69–75^. Further research is needed to clarify the association between elevated BP and neurodegeneration on a molecular level.

It is conceivable that the regulation of AH could be improved above the level achieved in RIFADE. Among others, 2 strategies appear promising. First, frequent use of home blood pressure measurement devices including applications for documentation of BP-values could provide a more detailed picture of the individual BP history and allow for a tighter circadian regulation of BP to normative values. This would be of particular importance for common cases of labile hypertension with high BP variability.

Second, national strategies to reduce sodium in nutrients should be intensified and prospectively evaluated due to methodologically incoherent results in former reports^76–82^. Moreover, educational campaigns should be enforced to increase awareness of the deleterious effects of increased sodium intake, in particular with regard to hypertension and cardiovascular disease, including hazardous effects apart from hypertension^83^. Finally, identifying and treating OSA will improve the effectiveness of treatment in hypertension and lead to a higher chance of favorable cognitive outcomes^84^.

It can be concluded that BP should be measured more frequently in subjects with cognitive complaints, not only to diagnose hypertension but also to monitor its correction status in order to avoid negative cognitive effects.

### Obstructive sleep apnea

OSA shows a significant effect on DemTect score as a single factor and in the 3-factorial analysis including AH and diagnosis as a factor. Results are in line with a small study on patients with moderate dementia, in which sufficient use of CPAP during a median time of 13.3 years led to a reduced amount of deterioration in global cognition measures and in improved executive functioning as compared to non-users of CPAP^37^. In a great study on the ADNI cohort, based on self-reported sleep apnea or obstructive sleep apnea, CPAP users with varying duration of CPAP use showed a delayed age at onset of MCI or Alzheimer’s dementia as compared to non-users of CPAP^47^. A French study on patients with mild to moderate AD found even stable or improved cognitive courses in 9 of 14 CPAP-treated OSA patients^46^. In MCI-patients, an increased psychomotor speed was observed in CPAP-users^31^. Recently Liguori et al.^48^ compared CPAP-adherent patients with non-adherent CPAP-users in a small cohort of patients with MCI or AD observing a smaller cognitive decline in the adherent group.

In sum, the current study is in accordance with findings of positive cognitive effects of OSA treatment in patients with NCD and is the first report on treated and untreated OSA patients in all stages of pre-existing neurocognitive disorder, showing favorable cognitive effects of treatment on the long-term in a substantial proportion of OSA patients.

Similar to the above-mentioned link between Alzheimer’s pathology and AH, there are also reports on associations of OSA with amyloid pathology^43,85^ contributing to the understanding of the pathophysiological consequences of OSA linked to hypoxia-induced dysfunctions^86^. Another aspect in this context is sleep fragmentation as an OSA-related factor resulting into cognitive decline^21^.

Whereas an exciting high proportion of patients with AH in this cohort received treatment (88%), only one-third of OSA patients got therapy, predominantly by CPAP, the gold standard of OSA treatment. This indicates a high need for intensified efforts in NCD patients with identified OSA to assess, whether CPAP treatment is applicable. On the other hand, it should be noted that a substantial amount of patients with untreated OSA had a favorable cognitive outcome. It is conceivable that a subgroup of OSA patients might have adapted to apnea-related hypoxia and other pathological events associated with OSA. Given the high proportion of patients, who do not tolerate CPAP treatment^87–89^, a search for biomarkers is required to estimate cognitive outcome in untreated OSA and the urgency of treatment at an individual level. In this context, it should be considered that recent reports point to a lack of erythrocytosis in OSA, which would be expected as an effect of chronic hypoxia^90^. This indicates, that markers of the hematopoietic system may not be suitable as such predictive biomarkers. Apart from these considerations, multidisciplinary efforts should be undertaken to reduce the prevalence of obesity as a main causal factor of OSA, which simultaneously helps to address a couple of other risk factors, e.g. hypertension.

### The glymphatic system as link of the risk factors OSA and AH

The term glymphatic is a neologism of ‘glio-lymphatic system’ and was coined by Iliff and Nedergaard^91^. In mice aquaporin-4 (AQP-4), water channels were found to be expressed at the endfeet of astrocytes. Usually, these endfeet enclose vasculature, arteries as well as veins, allowing an exchange of cerebrospinal (CSF) and interstitial (ISF) fluid thereby promoting the clearance of waste products from brain parenchyma (for review see^92,93^). This process is facilitated during sleep since interstitial space widens by about 60%^94^. CSF influx into the glymphatic system is dependent on arterial pulsation driven by respiratory and cardiac cycles. ISF efflux occurs along big sinuses and veins and finally drains into lymphatic vessels on the dura mater terminating the cervical lymph nodes^95,96^.

Two of the above expounded glymphatic features are particularly vulnerable to disturbances. Firstly, the CSF influx depends on arterial pulsation/cardiac cycle. In arterial hypertonus/AH there is a remodelling of the vascular wall leading to stiffness and reduced elasticity/pulsation. Impediments of pulsation in turn lead to decreased clearance of waste products from brain parenchyma/interstitial space^97^. The formation of ß amyloid plaques in Alzheimer’s disease has been shown to be promoted in AH. However longitudinal analysis of Danish patient registers revealed that high blood brain barrier permeable ß-blockers (Propranolol and Carvedilol) reduced the risk of Alzheimer’s disease compared to low permeable ß-blockers^98^. Treating AH may therefore be a preventive strategy to avoid later Alzheimer’s disease. This notion is supported by a recent meta-analysis of five randomized controlled trials showing reduced odds for getting Alzheimer’s disease in patients with blood pressure lowering medication^99^.

Secondly, sleep is facilitating the clearance of waste products from brain parenchyma. One night of sleep deprivation in healthy adults was shown to increase ß amyloid levels in a PET study^100^. Similarly, clearance of contrast agent gadobutrol was impaired in individuals with 24 h of sleep deprivation^101^. New diffusion tensor imaging studies using analysis along perivascular space (APLS) as measure of glymphatic functioning link both AH^102^ and OSA^103^ to glymphatic impairment. A recent study employing dynamic contrast enhanced MRI in OSA patients revealed enlarged perivascular spaces, lateral ventricles and disturbed glymphatic flow as well as cognitive deficits measured by MMST and MoCA^104^. After 1 month of CPAP glymphatic function of OSA patients improved, thus emphasizing the need of treating OSA as a risk factor for dementia/NCD.

### Arterial hypertension and obstructive sleep apnea

Following a concept of an integrated approach in the treatment of vascular risk factors, with a focus on endothelial dysfunction as a common final path resulting in malfunction of the neuro-vascular unit^105^, it seems obvious to consider hypertension and OSA together in the treatment of NCD. This is supported by the result in the current study, that patients who had treatment for both factors (AH × OSA) appear to have the best estimated marginal means for DemTect change (Table 4). The 2-factor analysis with AH and OSA as independent variables, however, revealed a non-significant result for both outcome variables. This could be mainly due to two reasons. First, the level of close adjustments of BP to normative values in RIFADE could be not as high as it could have been achievable. Moreover, the limited statistical power of RIFADE may have prevented significant results.

On the other hand, a synoptic view on cognitive outcome measures in OSA, which depend on the status of hypertension reveals interesting results (Fig. 2). DemTect change is best, if OSA *and* AH are treated. Untreated AH results in the worst cognitive outcome in OSA among all OSA states. In other words, an untreated hypertension status appears to reduce or even remove the effect of treatment in OSA. However, due to the small case numbers in this study, such results should be treated with caution and be verified in greater, well-balanced cohorts.

If the diagnosis is considered in analysis of the risk factors, a diagnosis-related pattern appears with regard to cognitive outcome (Supplementary Table 6). In all diagnostic groups, i.e. AD-NCD, vascular-NCD and mixed-NCD, the outcome is best, if OSA is treated and the outcome is better in treated compared to untreated groups with regard to OSA or AH. However, whereas in AD-NCD and vascular-NCD the combination of treated OSA and treated hypertension achieves the best results, in mixed-NCD this is the case in the combination with absent AH. On the other hand, the worst outcomes are obtained in combinations with untreated OSA in AD-NCD and vascular-NCD, in mixed-NCD this appears for the combination of absent OSA and untreated AH. Although caution is recommended to generalize those results due to small case numbers, those data point to a special relevance of hypertension in mixed-NCD. It is conceivable that the combination of Alzheimer’s and vascular pathology makes patients with mixed-NCD particularly susceptible to deleterious effects of untreated hypertension promoting cognitive decline.

Compared to other diagnostic groups, AD-NCD patients revealed the best cognitive outcome in the treated OSA condition averaged over all conditions of AH (treated, untreated, absent) (Table 5). Otherwise, in untreated OSA, AD-NCD showed the worst outcome compared to other diagnostic groups, when averaged over all AH conditions. This may indicate an essential effect of OSA in AD, which is in line with recent reports on favorable cognitive effects of OSA treatment in Alzheimer´s^48^.

For the group with vascular-NCD it can be stated, that cognitive *improvements* were observed in all conditions except for the untreated OSA combination with treated AH (Supplementary Table 6). However, it has to be considered, that the untreated condition for OSA *and* AH was not represented in this diagnostic group. Nevertheless, vascular-NCD reveals to be the most favorable condition with regard to the cognitive outcome as compared to AD-NCD and mixed-NCD (Table 5).

Another interesting observation was given by the analysis of diagnostic attributions in the group of untreated OSA patients. Namely, 90% of the patients with vascular-NCD and untreated OSA showed a favorable final cognitive outcome whereas this was only the case in 48% of the untreated OSA patients with mixed-NCD. This is in accordance with the above-mentioned approach, that in vascular patients treatment concepts should consider risk factors in an integrated view rather than treating each factor isolated. On the other hand, treatment of OSA appears all the more important, when AD pathology comes into play in form of mixed-NCD.

As mentioned elsewhere^50^, RIFADE has several limitations. One of them is the small number of enrolled patients. This disadvantage is partially compensated for by repeated measurements, which allow the analysis of both short-term and long-term effects of predictors such as risk factors. While it has been widely assumed in research for decades, that vascular risk factors, for example, only have an effect on the long term, the current data show that factors like hypertension may also exert cognotropic effects over shorter periods of time, from months to a few years.

As the main conclusion, it is stated that treatment of two risk factors contributed to favorable effects on cognitive outcomes in patients suffering from both most common neurocognitive disorders. Patients with AD- or vascular- / mixed-NCD should be screened for hypertension and obstructive sleep apnea and treated for these factors as far as possible. Efforts by health organizations such as the Berlin manifesto are helpful in putting this into action^106^.

## Supplementary Information

Supplementary Tables.

## Data Availability

The datasets generated during and/or analysed during the current study are not publicly available due to patient confidentiality but are available from the corresponding author on reasonable request.
