# Supplementary material for: Treatment of hypertension and obstructive sleep apnea counteracts cognitive decline in common neurocognitive disorders in diagnosis-related patterns
Source: Sci Rep. 2023 May 9;13:7556. doi: 10.1038/s41598-023-33701-2 (PMC10169815; doi:10.1038/s41598-023-33701-2)
Supplement: Supplementary file 1 — Supplementary Tables. [file 41598_2023_33701_MOESM1_ESM.docx]

**Supplementary Information**

Supplementary Table 1

*(Table 3.2b) Model 2: OSA as single factor*, Mixed linear model analysis of repeated DemTect scores as Dependent Variable with Mean Values for Risk factors in Different States

| OSA | M | SE | Df | Confidence interval 95% | |
| --- | --- | --- | --- | --- | --- |
|  |  |  |  | Lower Bound | Upper Bound |
| Absent | 11.386^a^ | 0.311 | 135.317 | 10.772 | 12.000 |
| Treated | 14.357^a^ | 0.669 | 219.193 | 13.039 | 15.675 |
| Untreated | 11.586^a^ | 0.488 | 158.625 | 10.622 | 12.550 |
| Note: *^a^* Covariates in the model were calculated using: Age 72.932, Education 1.45, Time since inclusion 973.1284, DemTect score baseline 11.993. For other abbreviations see Table 3.1  Supplementary Table 2  *(Table 3.4a) Model 4: AH x OSA x Diagnosis, Mixed Linear Model Analysis of Repeated DemTect Scores as Dependent Variable with Fixed Effects Type III*   \| Source \| Numerator-df \| Denominator-df \| F \| P \| \| --- \| --- \| --- \| --- \| --- \| \| Constant term \| 1 \| 124.788 \| 24.858 \| .000 \| \| Diagnosis \| 2 \| 123.538 \| 2.941 \| .057 \| \| AH \| 2 \| 170.676 \| 0.109 \| .897 \| \| OSA \| 2 \| 262.129 \| 6.141 \| .002 \| \| AH x OSA \| 3 \| 230.758 \| 0.843 \| .471 \| \| Diagnosis x AH \| 2 \| 110.821 \| 1.576 \| .211 \| \| Diagnosis x OSA \| 4 \| 222.028 \| 0.863 \| .487 \| \| Diagnosis x AH x OSA \| 2 \| 202.324 \| 3.329 \| .038 \| \| Age (yrs) \| 1 \| 134.785 \| 10.739 \| .001 \| \| Education \| 1 \| 105.786 \| 0.002 \| .962 \| \| Time \| 1 \| 347.829 \| 34.008 \| .000 \| \| DemTect baseline \| 1 \| 112.004 \| 111.371 \| .000 \| \| *Diagnosis* Diagnosis including: *AD* neurocognitive disorder (NCD) due to Alzheimer's disease, *MIXED* mixed-NCD, *VASC* vascular-NCD. For abbreviations see Table 3.1 \| \| \| \| \|   Supplementary Table 3  *(Table 3.4b) Model 4: AH x OSA x Diagnosis, Mixed linear model analysis of repeated DemTect scores as Dependent Variable with Mean Values for Risk factors in Different States*   \| OSA \| AH \| Diagnosis \| \| M \| SE \| Df \| Confidence interval 95% \| \| \| --- \| --- \| --- \| --- \| --- \| --- \| --- \| --- \| --- \| \|  \|  \|  \| \| Lower Bound \| Upper Bound \| \| Absent \| Absent \| \| AD \| 8.027^b^ \| 2.323 \| 86.883 \| 3.410 \| 12.643 \| \| MIXED \| 11.044^b^ \| 0.623 \| 130.348 \| 9.811 \| 12.277 \| \| VASC \| 12.950^b^ \| 1.305 \| 215.175 \| 10.378 \| 15.523 \| \| Treated \| \| AD \| 11.360^b^ \| 1.363 \| 129.670 \| 8.663 \| 14.056 \| \| MIXED \| 11.168^b^ \| 0.390 \| 126.554 \| 10.396 \| 11.939 \| \| VASC \| 13.244^b^ \| 0.840 \| 144.478 \| 11.583 \| 14.904 \| \| Untreated \| \| AD \| ^b,c^ \|  \|  \|  \|  \| \| MIXED \| 10.297^b^ \| 1.270 \| 356.196 \| 7.798 \| 12.795 \| \| VASC \| ^b,c^ \|  \|  \|  \|  \| \| Treated \| Absent \| \| AD \| ^b,c^ \|  \|  \|  \|  \| \| MIXED \| 16.506^b^ \| 1.211 \| 206.887 \| 14.119 \| 18.893 \| \| VASC \| 13.979^b^ \| 1.502 \| 135.386 \| 11.009 \| 16.950 \| \| Treated \| \| AD \| 15.341^b^ \| 3.090 \| 232.612 \| 9.254 \| 21.429 \| \| MIXED \| 11.017^b^ \| 1.305 \| 118.846 \| 8.434 \| 13.601 \| \| VASC \| 15.364^b^ \| 1.783 \| 107.143 \| 11.829 \| 18.899 \| \| Untreated \| \| AD \| ^b,c^ \|  \|  \|  \|  \| \| MIXED \| ^b,c^ \|  \|  \|  \|  \| \| VASC \| ^b,c^ \|  \|  \|  \|  \| \| Untreated \| Absent \| \| AD \| ^b,c^ \|  \|  \|  \|  \| \| MIXED \| 10.450^b^ \| 1.341 \| 234.944 \| 7.809 \| 13.092 \| \| VASC \| 13.258^b^ \| 1.274 \| 204.176 \| 10.747 \| 15.770 \| \| Treated \| \| AD \| 8.160^b^ \| 3.088 \| 232.549 \| 2.077 \| 14.243 \| \| MIXED \| 11.561^b^ \| 0.616 \| 130.641 \| 10.342 \| 12.780 \| \| VASC \| 12.812^b^ \| 0.976 \| 216.229 \| 10.888 \| 14.735 \| \| Untreated \| \| AD \| ^b,c^ \|  \|  \|  \|  \| \| MIXED \| 11.148^b^ \| 1.609 \| 356.670 \| 7.984 \| 14.312 \| \| VASC \| ^b,c^ \|  \|  \|  \|  \| \| Note: *^b^* Covariates in the model were calculated using: Age 73.505, Education 1.44, Time since inclusion 971.8319, DemTect score baseline 11.903. \| \| \| \| \| \| \| \| \| \| ^c^ Stepwise combination of factors was not observable. Marginal means not estimable  For other abbreviations see Table 3.1 and 3.4a \| \| \| \| \| \| \| \| \| | | | | | |

Supplementary Table 4

*(Table 4.1b) Model 1: AH as Single Factor,* Mixed linear model analysis of DemTect change as Dependent Variable with Mean Values for Risk Factors in Different States

| AH | M | SE | Df | Confidence interval 95% | |
| --- | --- | --- | --- | --- | --- |
|  |  |  |  | Lower Bound | Upper Bound |
| Absent | -.119^d^ | 0.312 | 366 | -0.734 | 0.495 |
| Treated | -.089^d^ | 0.188 | 366 | -0.459 | 0.281 |
| Untreated | -2.352^d^ | 0.821 | 366 | -3.966 | -0.737 |
| Note: *^d^* Covariates in the model were calculate using: Age 72.932, Education 1.45, Time between measurements/neurocognitive time (NCT) 428.8415, DemTect baseline 11.993. For other abbreviations see Table 3.1 | | | | | |

Supplementary Table 5

*(Table 4.3b) Model 3: AH x OSA, Mixed linear model analysis of DemTect change as Dependent Variable with Mean Values for Risk Factors in Different States*

| OSA | AH | M | SE | Df | Confidence interval 95% | |
| --- | --- | --- | --- | --- | --- | --- |
|  |  |  |  |  | Lower Bound | Upper Bound |
| Absent | Absent | -.212^d^ | 0.388 | 366 | -0.975 | 0.551 |
|  | Treated | .003^d^ | 0.244 | 366 | -0.477 | 0.483 |
|  | Untreated | -2.704^d^ | 1.184 | 366 | -5.032 | -0.376 |
| Treated | Absent | .264^d^ | 0.632 | 366 | -0.979 | 1.507 |
|  | Treated | .816^d^ | 0.698 | 366 | -0.556 | 2.188 |
|  | Untreated | -3.036^d^ | 1.806 | 366 | -6.587 | 0.516 |
| Untreated | Absent | -.207^d^ | 0.734 | 366 | -1.650 | 1.236 |
|  | Treated | -.463^d^ | 0.319 | 366 | -1.089 | 0.164 |
|  | Untreated | -1.190^d^ | 1.441 | 366 | -4.024 | 1.643 |
| For abbreviations and notes see Table 3.1 and 4.1 | | | | | | |

Supplementary Table 6

*(Table 6) OSA x AH x Diagnosis,*

| **OSA** | AH | | Diagnosis | | M | SE | Df | Confidence interval 95% | |
| --- | --- | --- | --- | --- | --- | --- | --- | --- | --- |
|  |  | |  | |  |  |  | Lower Bound | Upper Bound |
| Absent | | Absent | | AD | -.443^d^ | 1.405 | 366 | -3.207 | 2.320 |
|  |  |  |  | MIXED | -.430^d^ | 0.422 | 366 | -1.260 | 0.400 |
|  |  |  |  | VASC | 1.252^d^ | 1.089 | 366 | -0.890 | 3.394 |
|  |  | Treated | | AD | -.023^d^ | 0.985 | 366 | -1.959 | 1.913 |
|  |  |  |  | MIXED | -.117^d^ | 0.264 | 366 | -0.637 | 0.402 |
|  |  |  |  | VASC | .948^d^ | 0.636 | 366 | -0.303 | 2.198 |
|  |  | Untreated | | AD | ^c,d^ |  |  |  |  |
|  |  |  |  | MIXED | -2.202^d^ | 1.256 | 366 | -4.672 | 0.268 |
|  |  |  |  | VASC | ^c,d^ |  |  |  |  |
| Treated | | Absent | | AD | ^c,d^ |  |  |  |  |
|  |  |  |  | MIXED | .379^d^ | 0.861 | 366 | -1.313 | 2.071 |
|  |  |  |  | VASC | -.070^d^ | 0.917 | 366 | -1.874 | 1.733 |
|  |  | Treated | | AD | 7.622^d^ | 2.810 | 366 | 2.095 | 13.148 |
|  |  |  |  | MIXED | -.589^d^ | 0.913 | 366 | -2.385 | 1.207 |
|  |  |  |  | VASC | 2.244^d^ | 1.175 | 366 | -0.067 | 4.554 |
|  |  | Untreated | | AD | ^c,d^ |  |  |  |  |
|  |  |  |  | MIXED | ^c,d^ |  |  |  |  |
|  |  |  |  | VASC | ^c,d^ |  |  |  |  |
| Untreated | | Absent | | AD | ^c,d^ |  |  |  |  |
|  |  |  |  | MIXED | -1.366^d^ | 1.035 | 366 | -3.402 | 0.670 |
|  |  |  |  | VASC | .827^d^ | 0.994 | 366 | -1.128 | 2.781 |
|  |  | Treated | | AD | -4.446^d^ | 2.809 | 366 | -9.970 | 1.078 |
|  |  |  |  | MIXED | -.514^d^ | 0.344 | 366 | -1.191 | 0.163 |
|  |  |  |  | VASC | -.058^d^ | 0.729 | 366 | -1.492 | 1.376 |
|  |  | Untreated | | AD | ^c,d^ |  |  |  |  |
|  |  |  |  | MIXED | -1.895^b^ | 1.626 | 366 | -5.093 | 1.302 |
|  |  |  |  | VASC | ^c,d^ |  |  |  |  |
| Note: *^d^* Covariates in the model were calculated using: Age 72.932, Education 1.45, Time between measurements/neurocognitive time (NCT) 428.8415, DemTect score baseline 11.993. For other abbreviations see Tables 3.1 and 3.4 | | | | | | | | | |
